# Deacidification by FhlA-dependent hydrogenase is involved in urease activity and urinary stone formation in uropathogenic *Proteus mirabilis*

**DOI:** 10.1038/s41598-020-76561-w

**Published:** 2020-11-11

**Authors:** Wen-Yuan Lin, Shwu-Jen Liaw

**Affiliations:** 1 Department and Graduate Institute of Clinical Laboratory Sciences and Medical Biotechnology, College of Medicine, National Taiwan University, No. 1, Chang-Te Street, Taipei, 10016 Taiwan, ROC; 2 Department of Laboratory Medicine, National Taiwan University Hospital, National Taiwan University, Taipei, Taiwan, Republic of China

**Keywords:** Molecular biology, Pathogenesis

## Abstract

*Proteus mirabilis* is an important uropathogen, featured with urinary stone formation. Formate hydrogenlyase (FHL), consisting of formate dehydrogenase H and hydrogenase for converting proton to hydrogen, has been implicated in virulence. In this study, we investigated the role of *P. mirabilis* FHL hydrogenase and the FHL activator, FhlA*. fhlA* and *hyfG* (encoding hydrogenase large subunit) displayed a defect in acid resistance. *fhlA* and *hyfG* mutants displayed a delay in medium deacidification compared to wild-type and *ureC* mutant failed to deacidify the medium. In addition, loss of *fhlA* or *hyfG* decreased urease activity in the pH range of 5–8. The reduction of urease activities in *fhlA* and *hyfG* mutants subsided gradually over the pH range and disappeared at pH 9. Furthermore, mutation of *fhlA* or *hyfG* resulted in a decrease in urinary stone formation in synthetic urine. These indicate *fhlA*- and *hyf*-mediated deacidification affected urease activity and stone formation*.* Finally, *fhlA* and *hyfG* mutants exhibited attenuated colonization in mice*.* Altogether, we found expression of *fhlA* and *hyf* confers medium deacidification via facilitating urease activity, thereby urinary stone formation and mouse colonization. The link of acid resistance to urease activity provides a potential strategy for counteracting urinary tract infections by *P. mirabilis*.

## Introduction

Urinary tract infections (UTIs) are common infectious diseases, afflicting a large proportion of the human population. An estimated 40–50% of women will experience at least one UTI during their lifetime^1,2^. UTIs with usage of indwelling catheters and formation of urinary stones usually cause therapeutic failure^3–5^. Catheter-associated urinary tract infections (CAUTIs) are among the most common nosocomial infections, causing an enormous health and financial burden worldwide. In addition, the rise of antimicrobial resistance in uropathogens has complicated the treatment of UTIs^2,4,6^. Thus, new antibacterial strategies are open for further investigation.

Formate hydrogenlyase activator (FhlA) belongs to one of the RpoN-dependent enhancer binding proteins and is a master regulator of formate hydrogenlyase (FHL) which consists of formate dehydrogenase H (FDH), and hydrogenase multimers, connecting formate oxidation to proton reduction responsible for the H_2_ production^7–11^. Hydrogenases are enzymes that catalyze the oxidation of molecular hydrogen (H_2_) into protons or the reduction of protons to molecular hydrogen^12^. The hydrogenase of FHL belongs to [NiFe] hydrogenase involving in converting proton to hydrogen^11,13^. *Escherichia coli* could produce four distinct [NiFe]-hydrogenase isoenzymes including hydrogenase-1 (Hya), hydrogenase-2 (Hyb), hydrogenase-3 (Hyc) and hydrogenase-4 (Hyf) encoded by *hya*, *hyb*, *hyc* and *hyf* operon respectively^11,13^. The homologous Hyc and Hyf are energy-converting hydrogenases conserved in gammaproteobacteria such as uropathogenic *E. coli* (UPEC) and *Klebsiella pneumoniae*^13^. Hyc and Hyf are made of several components such as electron transfer proteins, membrane-anchored proteins and catalytic hydrogenase subunits^11,14,15^. The transcriptions of both *hyc* and *hyf* operons are activated by FhlA and are dependent on RpoN^7–10^. Taking *E. coli* as an example, Hyc and Hyf would couple with formate dehydrogenase to form FHL-1 and FHL-2, respectively. At slightly acidic pH 6.5, the production of H_2_ was mostly dependent on FHL-1, while FHL-2 contributes to H_2_ production at slight alkaline pH 7.5^16^. The FHL-1 of *E coli* contributes to medium deacidification during mix acid fermentation^17^ and acid resistance in the anaerobic environment^18^. In addition, hydrogenases of FHL might play a role in virulence by neutralizing hydroxyl free radicals (OH·), providing energy and maintaining acid–base homeostasis^19^. In this regard, *fhlA* and FHL-2 genes have been shown to be induced in UPEC during urinary tract infections^20^. Recently, *fhlA* and the genes encoding the components of FHL-2, were found to be likely to contribute to mouse colonization in uropathogenic *Proteus mirabilis* by the genome-wide study^21^. In view that enzymes and the maturation machinery required for production of FHL hydrogenases are completely different from human proteins^22^, these pathways would be promising targets for the development of new antimicrobial strategies.

*Proteus mirabilis* is an important pathogen of the urinary tract, especially in patients with indwelling urinary catheters^5^. UTIs and CAUTIs involving *P. mirabilis* are typically complicated by the formation of bladder and kidney stones due to alkalinization of urine from urease-catalyzed urea hydrolysis, leading to catheter blockage and permanent renal damage^3^. In addition, urinary stones deposit on the catheter surface, facilitating the formation of crystalline biofilms^3^. On the basis of the genome-wide study by Armbruster et al.^21^, we investigated if FhlA and FHL hydrogenase are associated with virulence factor expression of *P. mirabilis*. Here, we show that FhlA-regulated hydrogenase gene expression accelerated medium deacidification, whereby facilitating urease activity and urinary stone formation. Moreover, we confirmed the fitness defect of *fhlA* and hydrogenase gene (*hyfG*) mutants in UTI mouse model of *P. mirabilis*. This is the first report revealing *P. mirabilis fhlA* and hydrogenase genes participate in urease activity, urinary stone formation and virulence. This finding provides a perspective for development of new therapeutic approaches to counteract UTIs caused by *P. mirabilis*.

## Results

### Counterparts of the FHL hydrogenase *hyc* locus, *fhlA* and *fdhF* genes in *P. mirabilis* N2

**Figure 1:**
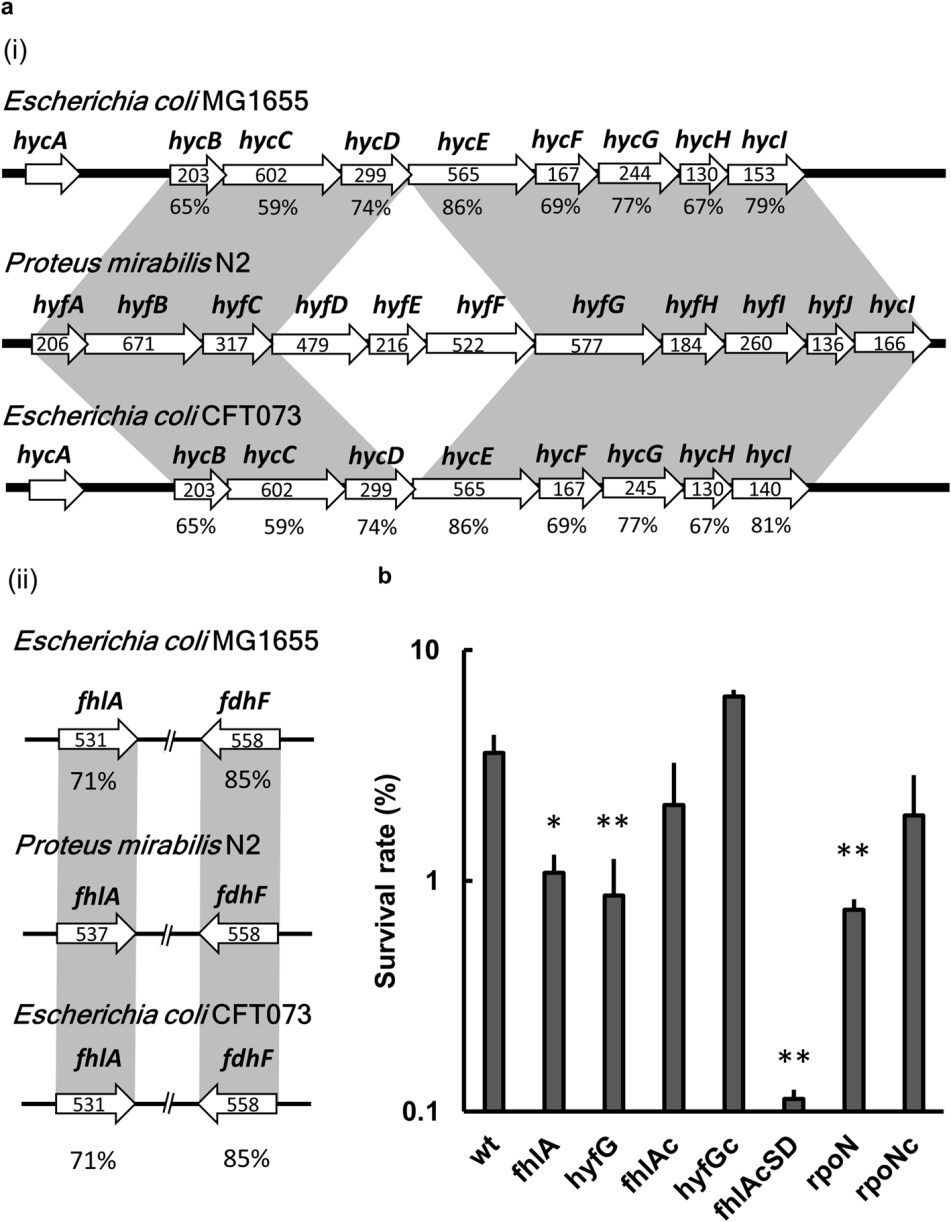
*P. mirabilis hyf* locus and involvement of FHL-related genes in acid resistance. (**a**) (i) The *P. mirabilis* FHL hydrogenase gene locus (*hyfA-J-hycI*) corresponds to the well characterized *hyc* locus in *E. coli* K-12 MG1655 and UPEC CFT073 with corresponding genes in shadows. An amino acid sequence analysis of the *hyf* locus in *P*. *mirabilis* N2 and its counterparts in *E*. *coli* K-12 MG1655 and *E*. *coli* CFT073 was performed using position-specific iterative BLAST. The eight proteins of the *hyf* locus in *P*. *mirabilis* N2 are similar to the HycB, HycC, HycD, HycE, HycF. HycG, HycH and HycI in *E*. *coli* K-12 MG1655 and *E*. *coli* CFT073. The percent amino acid similarities between *P*. *mirabilis* N2 and *E*. *coli* K-12 MG1655 or *E*. *coli* CFT073 were shown below each gene. The number inside white arrow represents the amino acid length of each protein. (ii) The *P*. *mirabilis fhlA* and *fdhF* loci correspond to those of *E. coli* K-12 MG1655 and UPEC CFT073 with corresponding genes in shadows. An amino acid sequence analysis was carried out the same as above. The predicted amino acid sequences of *fhlA* and *fdhF* in *P*. *mirabilis* N2 are similar to FhlA and formate dehydrogenase-H respectively in *E*. *coli* K-12 MG1655 and *E*. *coli* CFT073. The percent amino acid similarities between *P*. *mirabilis* N2 and *E*. *coli* K-12 MG1655 or *E*. *coli* CFT073 were shown below each gene. The number inside white arrow represents the amino acid length of each protein. (**b**) The survival rate of the wild-type, mutants (*fhlA, hyfG* and *rpoN*), complemented strains (*fhlA*c*, hyfG*c and *rpoN*c) and *fhlA*cSD strain after exposure to acid (pH 3). Acid survival rate (expressed as percent) was calculated with the following formula: 100 × (CFU after acid treatment/CFU before acid treatment). Significant differences from the wild-type bacteria were determined by using the one-way ANOVA with Tukey’s multiple-comparison test (*P < 0.05, **P < 0.01). The data are averages and standard deviations of three independent experiments. wt, wild-type; fhlA, *fhlA* mutant; hyfG, *hyfG* mutant; fhlAc, *fhlA* complemented strain; hyfGc, *hyfG* complemented strain; fhlAcSD, *fhlA* mutant containing altered FhlA (F291I, T292S) in pGEM-T Easy; rpoN, *rpoN* mutant; rpoNc, *rpoN* complemented strain.

To investigate the role of FhlA and FHL in uropathogenic *P. mirabilis*, we searched the genome of *P. mirabilis* HI4320 for the homologous genes of FhlA, FHL hydrogenase and formate dehydrogenase H (FDH-H encoded by *fdhF*). Only a putative FHL hydrogenase 4 operon (*hyf*) is present in *P. mirabilis* HI4320. No similar FHL hydrogenase 3 operon (*hyc*) was found in *P. mirabilis* HI4320. We amplified the entire DNA fragment (11,956 bp) containing *hyfABCDEFGHIJhycI* and the upstream region of *hyfA* from the genomic DNA of *P. mirabilis* N2 using the KOD DNA polymerase of high fidelity and efficiency by primers designed from *P. mirabilis* HI4320 (Table 1) and the sequence of the product was determined. *hyfA* and *hyfH* may encode electron carrier proteins containing 4Fe-4S domain; *hyfB*, *hyfD* and *hyfF* may encode proton-conducting membrane transporters; *hyfC* and *hyfE* may encode membrane-anchored subunits; *hyfG* and *hyfI* may encode [NiFe] hydrogenase large and small subunits respectively; *hyfJ* and *hycI* may encode proteins for maturation of HyfG. As shown in Fig. 1a, the *P. mirabilis* N2 *hyf* locus consists of *hyfABCDEFGHIJhycI*. The putative proteins encoded by *hyf* locus of *P. mirabilis* N2 shared 59–86% similarities with their respective orthologues in *E. coli* MG1655 and CFT073 respectively, with highest similarity between hydrogenase large subunit (HyfG vs HycE) (Fig. 1a). The nucleotide sequences of *fhlA* (2572 bp) and *fdhF* (2810 bp) containing the upstream promoter region were also acquired by amplification with primers designed from *P. mirabilis* HI4320 (Table 1) and sequencing the product. The BLAST search revealed FhlA and FDH-H orthologues with 71% and 85% similarities to that of uropathogenic *E. coli* CFT073 and nonpathogenic *E. coli* MG1655 respectively (Fig. 1a). The nucleotide sequences of *fhlA*, *hyf* locus and *fdhF* were assigned accession number MT492462, MT492463 and MT492464 respectively by the GenBank database.

**Table 1:** Primers used in this study.

Primer	Sequence (5′–3′)	Description
*fhlA*_com_F	GGATCCATAAGAAAATAGCGGTTTGG	For *fhlA* complementation. Paired with “*fhlA*_com_R”
*fhlA*_com_R	GTCGACCTAAAGAATTTCAGGAATGGA	For *fhlA* gene locus sequencing. Paired with “*fhlA*_promoter_F”
*hyfG*_com_F	TCGGTTCAGGAGAAGTAACG	For *hyfG* complementation. Paired with “*hyfG*_com_R”
*hyfG*_com_R	CTCACTTGAGCGGCGAAT	
*rpoN*_com_F	GAG CTCAAGAGGATCCATCGCATC	For *rpoN* complementation. Paired with “*rpoN*_com_R”
*rpoN*_com_R	CTCGAGCCATAGTGTCTTCCTTCT	
*hyfA*_promoter_F	GCATGCAGATCCTTCAGGTTATG	For *hyfA* reporter assay. Paired with “*hyfA*_promoter_R”
*hyfA*_promoter_R	CTGCAGTCGCCACATCCTCCGAGA	
*fhlA*_promoter_F	TTAGAACAATAAATCAGCG	For *fhlA* reporter assay. Paired with “*fhlA*_promoter_R”
*fhlA*_promoter_R	TCTAGACATGACACCACCGATTGC	
*fdhF*_promoter_F	GCATGCGGCACACAGAATTCTCACAA	For *fdhF* reporter assay. Paired with “*fdhF*_promoter_R”
*fdhF*_promoter_R	CTGCAGCAACATTGCTCCTGTGTTCA	
*fdhF*_R	TTAGCCTTCAGCGGCTTCTCTTA	For *fdhF* gene locus sequencing. Paired with “*fdhF*_promoter_F”
*hyf*_R	CTATTCTTCGATATCGTCTTC	For *hyf* operon genes locus sequencing. Paired with “*hyfA*_promoter_F”
*fhlA*_site_direct_F	CGGGTGCAACACATGCCC	For *fhlA*cSD strain construction. Paired with “*fhlA*_site_direct_R”
*fhlA*_site_direct_R	AAATAGCGCCTTTATCATGTCC	

The underlined sequences represent restriction endonuclease cutting sites.

### *fhlA* and *hyf* are involved in acid resistance

Knowing FhlA is the transcriptional activator for the expression of the genes encoding FHL components of *E. coli*^8,10^ and *E. coli hycE* (encoding catalytic large subunit of hydrogenase 3) mutant has been shown to display reduced acid survival compared to the wild-type strain^18^, we first investigated whether FhlA and Hyf hydrogenase affect acid resistance in *P. mirabilis* N2. We performed homologous recombination to construct kanamycin-resistant *fhlA* and *hyfG* mutants, followed by Southern blotting to verify the mutant clone. In view that the large subunit of hydrogenase is critical for hydrogenase activity^18^, *hyfG* deletion stands for functional loss of Hyf activity in the study. There was no difference between the growth of the wild-type bacteria and respective mutants (Fig. 6c). We then tested the ability of the wild-type strain, *fhlA* and *hyfG* mutants and respective complemented strains to survive the acid exposure. We found that the survival rate of both *fhlA* and *hyfG* mutants was significantly lower than that of the wild-type strain and the respective complemented strains (Fig. 1b). The results indicate that FhlA and Hyf are involved in resistance to extreme acid exposure in *P. mirabilis*. FhlA was reported as an enhancer binding protein which could hydrolyze ATP to activate RpoN-dependent transcriptions through the RpoN-interacting motif, GAFTGA^23^. Thus, we constructed the site-directed mutant, *fhlA*cSD strain (the *fhlA* mutant containing an altered FhlA with the conserved RpoN-interacting motif GAFTGA changing into GAISGA in the pGEM-T Easy vector) which exhibited no growth defect compared to the wild-type bacteria. Then we assessed the survival in acid using the *fhlA*cSD strain, *rpoN* mutant and the *rpoN*-complemented strain. The survival of *fhlA*cSD strain and *rpoN* mutant was reduced relative to the wild-type and the *rpoN*-complemented strain. We concluded that FhlA (requiring the GAFTGA motif for RpoN-interaction), RpoN and HyfG contribute to the acid resistance (Fig. 1b).

### *hyf* and *fdhF* are regulated by FhlA and induced by formate and anaerobiosis

**Figure 2:**
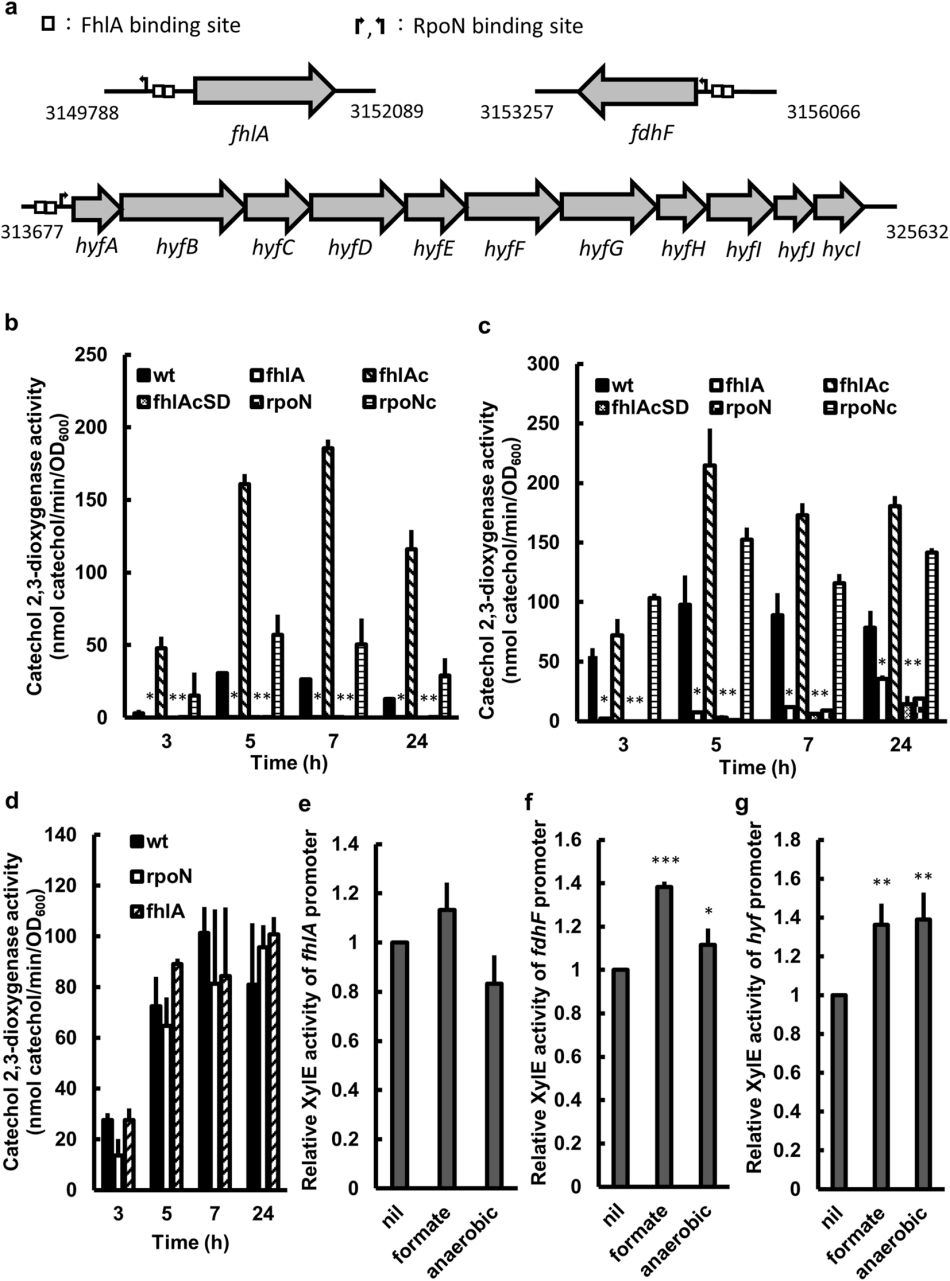
The transcription regulations of *hyf*, *fdhF* and *fhlA* genes in *P. mirabilis*. (**a**) Schematic promoter region of *fhlA, fdhF* and *hyf*. FhlA and RpoN binding sites were predicted by Regulatory Sequence Analysis Tools^24^. Promoter activities of (**b**) *hyf*, (**c**) *fdhF* and (**d**) *fhlA* in wild-type, mutants (*fhlA* and *rpoN*), complemented strains (*fhlA*c and *rpoN*c) and *fhlA*cSD strain. The activity of XylE in the *hyf-*, *fdhF-* or *fhlA-xylE* reporter plasmid-transformed bacterial strain was determined using the reporter assay at 3, 5, 7 and 24 h after incubation. (**e**–**g**) Inductivity of promoter activities of *fhlA, fdhF* and *hyf* in wild-type strain by formate or anaerobiosis. The overnight cultures of *fhlA-*, *fdhF-* or *hyf-xylE* reporter plasmid-transformed wild-type strain were regrown for 5 h. The activity of XylE in the reporter plasmid-transformed wild-type strain was determined after treatment of formate or anaerobiosis for 30 min. nil, aerobic condition; formate, 30 mM formate; anaerobic, anaerobic condition. Significant differences from the wild-type bacteria were determined by using two-way ANOVA (in **b**–**d**) and by one-way ANOVA (in **e**–**g**) with Tukey’s multiple-comparison test (*P < 0.05; **P < 0.01; ***P < 0.001). The data are averages and standard deviations of three independent experiments. wt, wild-type; fhlA, *fhlA* mutant; fhlAc, *fhlA* complemented strain; fhlAcSD, *fhlA* mutant altered FhlA (F291I, T292S) in pGEM-T Easy; rpoN, *rpoN* mutant; rpoNc, *rpoN* complemented strain.

In *E. coli*, the transcription of both *fdhF* and *hyf* operons is regulated by FhlA^8,10^. To identify conserved promoter elements for FhlA-dependent transcriptions, we submitted position weight matrices of FhlA and RpoN from characterized FhlA- and RpoN-dependent promoters (Table 2) to the Regulatory Sequence Analysis Tools (RSAT) sever^24^. TGGCACGNNNNTTGCA/T and the palindromic sequence TGA/TC-A/GAA/TA/GAT-GA/TCA were shown to be the conserved binding sites for RpoN and FhlA respectively. We found two putative FhlA and one RpoN conserved regulatory sequences present in the promoter regions of all the determined *fhlA*, *hyf* and *fdhF* sequences (Fig. 2a). We therefore performed the reporter assay to examine if *fhlA*, *hyf* and *fdhF* are regulated by FhlA and RpoN. We found *hyf* and *fdhF* promoter activities were reduced in the *fhlA* and *rpoN* mutants relative to the wild-type and respective complemented strains at 3, 5, 7, and 24 h after incubation (Fig. 2b,c). In addition, the RpoN-interacting domain of FhlA is responsible for regulation of *hyf* and *fdhF* promoter activities (Fig. 2b,c). According to the RpoN conserved regulatory sequence not in the *fhlA* gene direction, the reporter assay showed loss of *rpoN* or RpoN-dependent *fhlA* had no effect on *fhlA* promoter activity during the time period tested (Fig. 2d). It is known *fdhF*, *hyf* and *fhlA* could be induced by either formate or anaerobiosis in *E. coli*^8,10,25^. We tested the effect of 30 mM formate or anaerobic condition on the promoter activities of *fhlA*, *fdhF* and *hyf* after incubation for 5 h. We found *P. mirabilis fdhF* and *hyf* were induced significantly by 30 mM formate and anaerobiosis respectively (Fig. 2f,g) while formate and anaerobiosis seemed not have a significant effect on *fhlA* promoter activity (Fig. 2e). The results of *fhlA* promoter activity contrasted with previous reports that showed formate and anaerobiosis could trigger expression of FhlA-regulated *hypABCDE*-*fhlA* in *E. coli*^10,26^. Figure 2b–d showed the wild-type *P. mirabilis* displayed *hyf*, *fdhF* and *fhlA* promoter activities under the aerobic condition at 3, 5, 7 and 24 h after incubation. In summary, promoter activities of *fdhF* and *hyf* were under the control of FhlA in response to formate and anaerobiosis.

**Table 2:** Position-weight matrices of FhlA and RpoN binding sequences.

FhlA	1	2	3	4	5	6	7	8	9	10	11	12	13	14		
A	1	0	4	2	5	7	5	5	9	0	0	6	1	7		
C	0	1	1	10	2	2	1	0	1	0	2	1	11	1		
G	2	12	1	0	5	1	2	5	2	0	10	1	1	4		
T	10	0	7	1	1	3	5	3	1	13	1	5	0	2		

RpoN	1	2	3	4	5	6	7	8	9	10	11	12	13	14	15	16
A	12	2	0	12	139	11	55	51	46	44	38	13	4	1	9	76
C	14	0	0	147	23	122	17	48	64	42	62	22	18	2	173	5
G	10	184	186	6	18	10	103	69	36	35	43	15	10	181	1	17
T	150	0	0	21	6	43	11	18	40	65	43	136	154	2	3	88

These weight matrices based on 13 characterized FhlA-dependent promoters^10,59^ and 186 characterized RpoN-dependent promoters^60^.

### The deacidification of the medium requires *fhlA* and *hyf*

**Figure 3:**
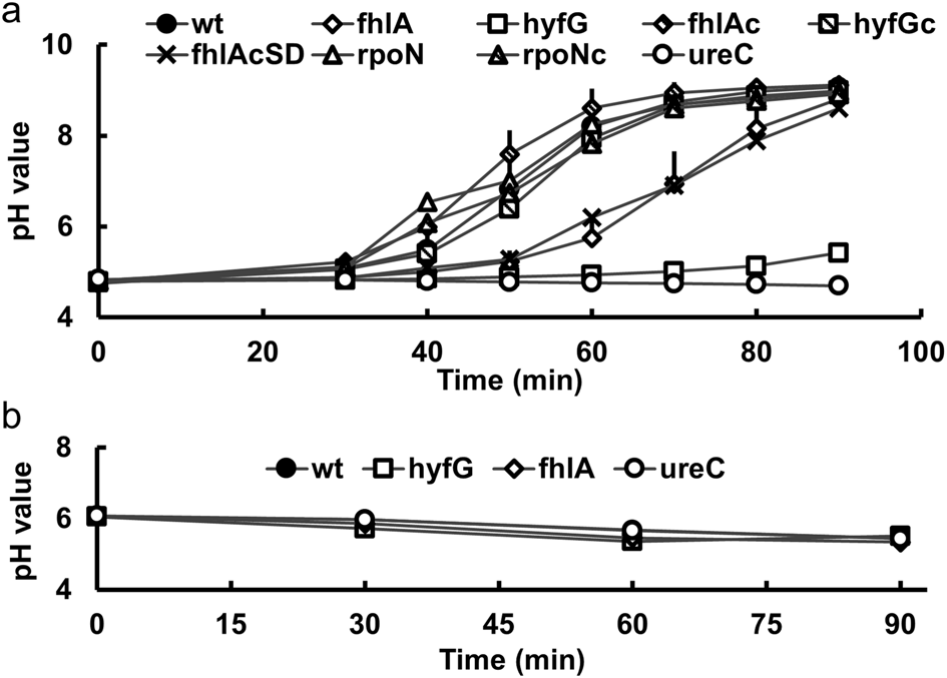
Urease activity and urea are required for *P. mirabilis* medium deacidification mediated by *fhlA* and *hyfG*. (**a**) Overnight cultures of the wild-type, mutants (*fhlA, hyfG*, *rpoN*, *fhlA*cSD and *ureC*) and complemented strains (*fhlA*c*, hyfG*c and *rpoN*c) were regrown for 5 h and resuspended in synthetic urine (pH 4.8 ± 0.2). The pH value was monitored during 30–90 min at 10-min intervals. (**b**) Overnight cultures of the wild-type and mutants of *fhlA, hyfG* and *ureC* were regrown for 5 h and resuspended in synthetic urine (pH 6.0 ± 0.2) in which the urea has been replaced by an equivalent amount of NH_4_Cl. The pH value was monitored at 30-min intervals over 90 min. The data are averages and standard deviations of three independent experiments. wt, wild-type; fhlA, *fhlA* mutant; hyfG, *hyfG* mutant; fhlAc, *fhlA* complemented strain; hyfGc, *hyfG* complemented strain; fhlAcSD, *fhlA* mutant containing altered FhlA (F291I, T292S) in pGEM-T Easy; rpoN, *rpoN* mutant; rpoNc, *rpoN* complemented strain; ureC*, ureC* mutant (without urease activity).

It has been known that formate, a product of mixed-acid fermentation, can be converted to H_2_ and CO_2_ by the FHL complex, in a process consuming protons and resulting in medium deacidification, thereby increasing bacterial survival^17^. We hypothesized that uropathogenic *P. mirabilis* could use the FHL system to consume protons, leading to deacidification of the environment. Therefore, we performed deacidification experiments using the synthetic urine at pH 4.8 (± 0.2) to test if loss of *hyfG* or *fhlA* affects the deacidification ability. The data showed both *hyfG* and *fhlA* mutants displayed a significant delay in deacidification, especially for *hyfG* mutant, compared to the wild-type and respective complemented strains (Fig. 3a). A similar reduction in deacidification ability of *fhlA* mutant was observed in the *fhlA*cSD mutant while *rpoN* mutant had a similar deacidification pattern to the wild-type strain (Fig. 3a).

### Urease activity of *P. mirabilis* is pH-dependent and loss of *hyf* or *fhlA* gene affects urease activity and urinary stone formation

**Figure 4:**
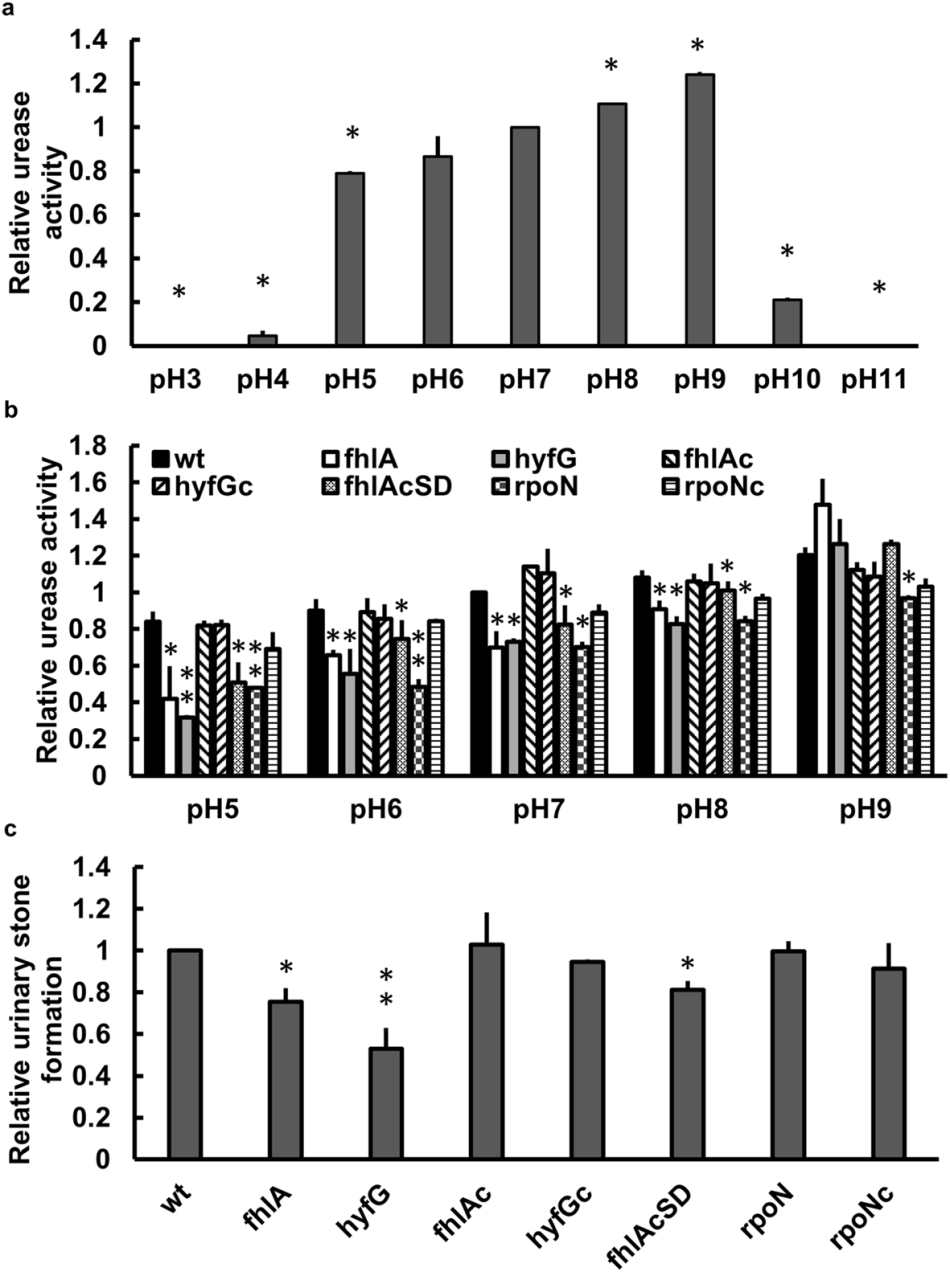
The pH-dependent urease activity and the effect of *fhlA*, *hyfG* or *rpoN* mutation on urease activity and stone formation in *P. mirabilis*. (**a**) The urease activity of wild-type bacteria regrown in LB for 5 h was determined by phenol-hypochlorite assay at pH 5–9. The relative urease activity is calculated with the following formula: OD_625_ of test/OD_625_ of wt at pH 7. Significant differences from the result at pH 7 were determined by using the Student’s *t* test (*P < 0.05). (**b**) Urease activities of the wild-type, mutants (*fhlA, hyfG* and *rpoN*), complemented strains (*fhlA*c*, hyfG*c and *rpoN*c) and *fhlA*cSD strain regrown in LB for 5 h were measured at different pH values. The relative urease activity is calculated with the following formula: OD_625_ of test/OD_625_ of wt at pH 7. Significant differences from the wild-type bacteria at each pH were determined by using two-way ANOVA with Tukey’s multiple-comparison test (*P < 0.05; **P < 0.01). (**c**) Stone formation of the wild-type, mutants (*fhlA, hyfG* and *rpoN*), complemented strains (*fhlA*c*, hyfG*c and *rpoN*c) and *fhlA*cSD strain. Bacteria were regrown and adjusted to 2 × 10^8^ CFU/ml with synthetic urine and incubated for 1 h at 37 ℃. The optical density at 630 nm of suspensions was measured as the intensity of stone formation. The relative urinary stone formation is calculated with the following formula: OD_630_ of test/ OD_630_ of wt. Significant differences from the wild-type bacteria were determined by using one-way ANOVA with Tukey’s multiple-comparison test (*P < 0.05; **P < 0.01). The data are averages and standard deviations of three independent experiments. wt, wild-type; fhlA, *fhlA* mutant; hyfG, *hyfG* mutant; fhlAc, *fhlA* complemented strain; hyfGc, *hyfG* complemented strain; fhlAcSD, *fhlA* mutant containing altered FhlA (F291I, T292S) in pGEM-T Easy; rpoN, *rpoN* mutant; rpoNc, *rpoN* complemented strain.

Urease can catalyze the hydrolysis of urea into ammonia, rising pH value and promoting urinary stone formation^27,28^. The urease activity changes with different pH value in *Helicobacter pylori*, being activated by acidic pH down to pH 2.5 and 3^29^. To understand whether urease activity of *P. mirabilis* is affected by pH value, we monitored the urease activity in LB broth at different pH values and found that urease activity was very low at pH 4, increased gradually in the pH range from 5 to 9 and dropped suddenly at pH 10 (Fig. 4a). Knowing *fhl*A and *hyfG* contributed to acid resistance, we assessed the urease activity in wild-type strain, *hyfG* and *fhl*A mutants and respective complemented strains in the pH range from 5 to 9. The data showed that urease activity of *hyfG* and *fhlA* mutants were lower than the wild-type and respective complemented strains in the pH range from 5 to 8 (Fig. 4b). Likewise, *fhlA*cSD strain and *rpoN* mutant had reduced urease activity relative to the wild-type bacteria in this pH range (Fig. 4b). The *rpoN* mutant could restore the activity to a certain level from pH 5 to 9 after *rpoN*-complementation (Fig. 4b). Interestingly, we found the urease activity of *hyfG*, *fhl*A and *fhlA*cSD mutants was not different from that of the wild-type strain at pH 9 while urease activity of *rpoN* mutant still decreased relative to the wild-type bacteria (Fig. 4b). The results show that both urease activity and the involvement of *fhl*A and *hyfG* in the urease activity is pH-dependent in *P. mirabilis*. Subsequently, we assessed the urinary stone formation using the same synthetic urine at pH 5.8. We found that the ability of stone formation in *fhlA* and *hyfG* mutants was significantly lower than that of wild-type strain and respective complemented strains (Fig. 4c). The ability to form urinary stones of *fhlA*cSD mutant significantly decreased relative to the wild-type, in contrast to that of *rpoN* mutant being comparable to the wild-type strain.

To differentiate between direct proton consumption and modulation of urease activity for the role of *hyf* and *fhlA* in medium deacidification, we included the *ureC* (encoding urease subunit) mutant in deacidification assay in synthetic urine. No medium deacidification but slight acidification by the *ureC* mutant was observed (Fig. 3a). In addition, using synthetic urine medium in which urea has been replace by an equivalent amount of NH_4_Cl, the medium deacidification did not occur in wild-type and mutants of *hyfG*, *fhlA* and *ureC* (Fig. 3b). Slight acidification was observed for wild-type and the mutants. The data suggest that urea hydrolyzed by urease is an essential process to deacidify synthetic urine medium for *P. mirabilis* and the role of *hyfG* and *fhlA* in medium deacidification is mainly dependent on modulation of urease activity. These results suggest that *fhlA*-regulated *hyf* expression could help *P. mirabilis* to deacidify the environment via facilitating urease activity and subsequent urinary stone formation.

### *fhlA*-regulated *hyf* expression assists bacterial colonization in the mouse urinary tract

**Figure 5:**
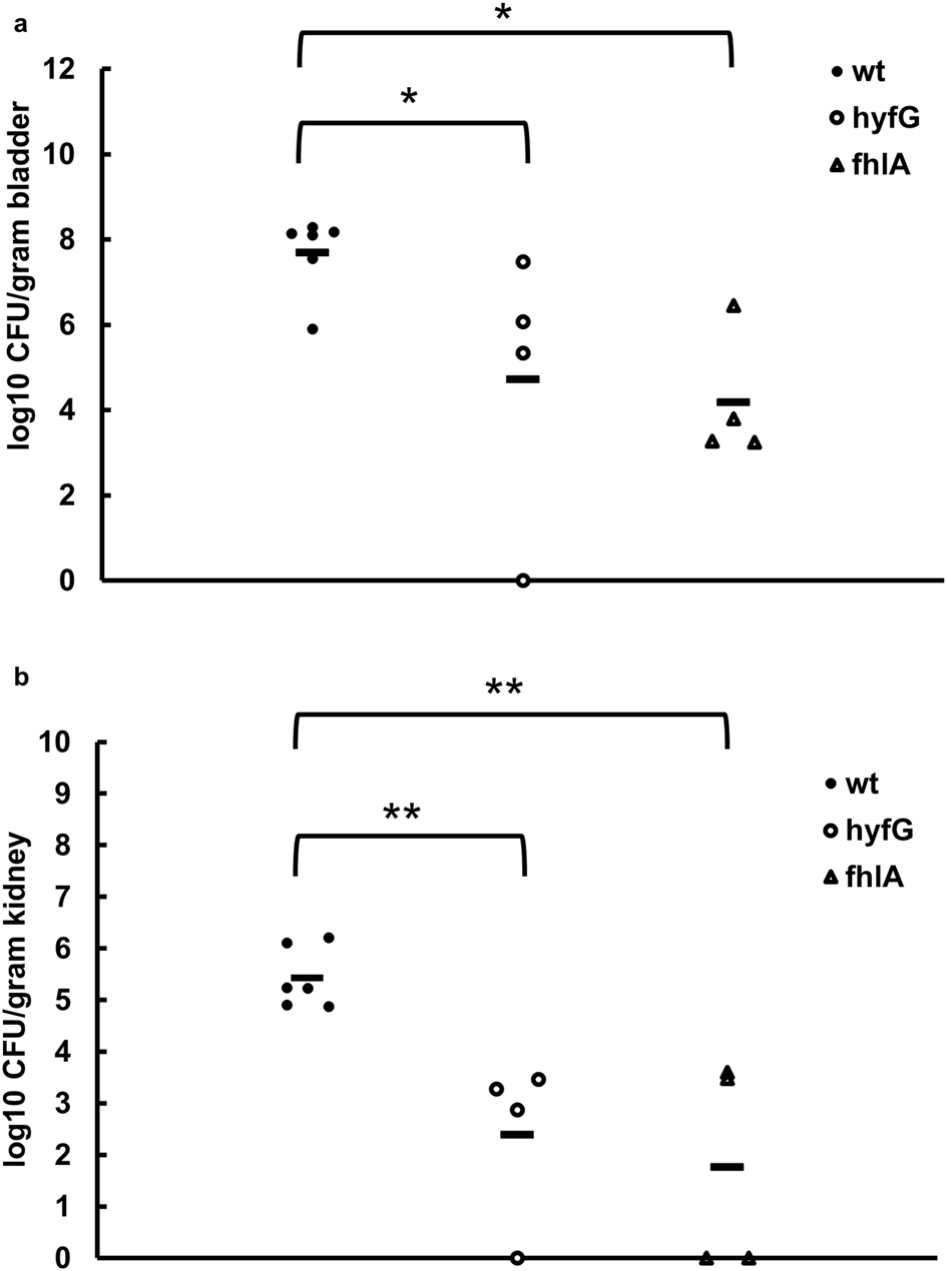
Colonization in mice by the wild-type *P. mirabilis*, *hyfG* mutant or *fhlA* mutant. ICR mice were inoculated transurethrally with bacteria at a dose of 1.5 × 10^7^ CFU per mouse. Bacterial loads (CFU) in the (**a**) bladders and (**b**) kidneys were determined on day 3 after inoculation. Horizontal bars indicate the average for each group, and the limit of detection was 100 CFU/g organ. Significant differences were determined using the Wilcoxon rank sum test (*P < 0.05; **P < 0.01). wt, wild-type; hyfG, *hyfG* mutant; fhlA, *fhlA* mutant.

To confirm the finding that FHL and FhlA were responsible for mouse colonization in *P. mirabilis*^21^, we investigated if *fhlA* and *hyfG* are associated with UTIs caused by *P. mirabilis.* The colonization ability was assessed in wild-type strain and mutants of *hyfG* and *fhlA* using the UTI mouse model^30^. In the bladder, the colonization ability of *fhlA* and *hyfG* mutant were significantly lower than wild-type strain (Fig. 5a). Both *fhlA* and *hyfG* mutants exhibited a significantly low ability of colonization in the kidney relative to the wild-type strain (Fig. 5b).

### Loss of *fhlA* or *hyf* does not affect proton motive force-related motility, drug susceptibility and growth under carbonyl cyanide m-chlorophenyl hydrazone (CCCP)

**Figure 6:**
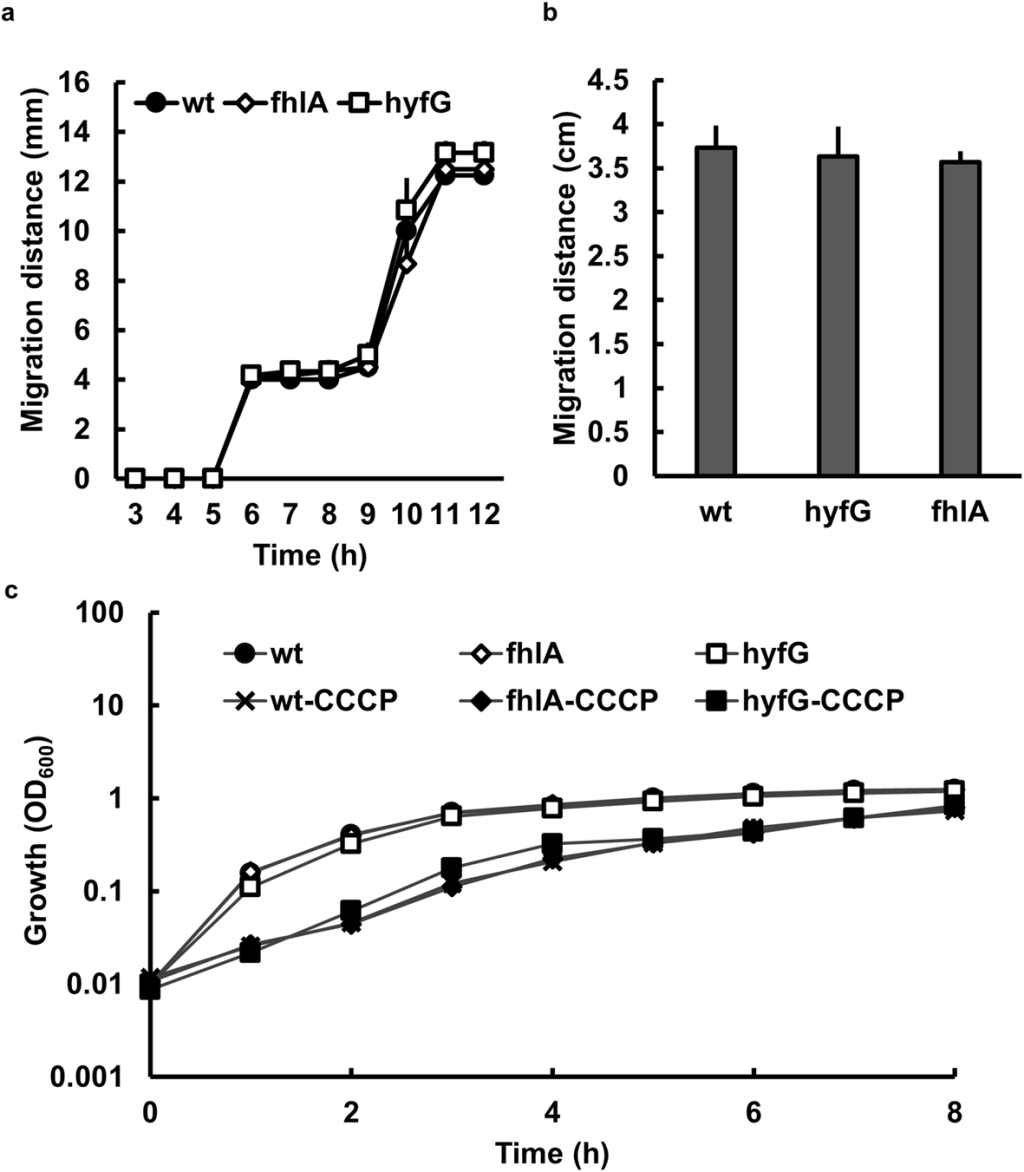
Proton motive force-related phenotypes in *P. mirabilis*. (**a**) The swarming migration distance of wild-type and *fhlA* and *hyfG* mutants was monitored by following swarm fronts of the bacterial cells and recording progress at 1-h intervals. (**b**) The swimming migration distance of wild-type and mutants of *fhlA* and *hyfG* was recorded at 18 h after inoculation. (**c**) Growth of the wild-type and mutants (*fhlA* and *hyfG*) cultured in LB broth with and without CCCP (7.5 μM) was monitored by measurement of OD_600_. The data are averages and standard deviations of three independent experiments. wt, wild type; fhlA, *fhlA* mutant; hyfG, *hyfG* mutant.

Previous studies showed that Hyf complex is similar to respiratory NADH dehydrogenase complex I^11,13,14^ and the complex I was shown to provide the proton motive force (PMF)^31^, which could affect acid resistance, susceptibilities of polymyxin B and aminoglycosides, motility and growth under treatment of the PMF uncoupler^32–37^. We monitored the PMF-related phenotypes including drug susceptibilities and motility in the wild-type bacteria and *fhlA* and *hyfG* mutants. Neither the MICs of the drugs (polymyxin B, streptomycin and gentamicin) used nor the swarming and swimming motility was different between wild-type strain and respective mutants (Table 3; Fig. 6a,b). We also examined the effect of CCCP, a PMF uncoupler, on growth of the wild-type, *hyfG* mutant and *fhlA* mutant. The growth curves of all strains showed no difference in the presence or the absence of CCCP (Fig. 6c). These results indicate that mutation of *fhlA* or *hyfG* did not affect the PMF-related phenotypes examined except acid resistance. It implies that the consumption of protons may be the main reason for FhlA-regulated *hyf* expression in acid resistance.

**Table 3:** Minimal inhibitory concentration of gentamicin (Gm), polymyxin B (PB) and streptomycin (Sm) in wild-type (wt) and mutants (*hyfG* and *fhlA*).

Strain	MIC (μg/ml)		
	PB	Gm	Sm
wt	50,000	8	32
*hyfG*	50,000	8	32
*fhlA*	50,000	8	32

## Discussion

To our knowledge, this study is the first to demonstrate that RpoN-dependent FhlA and hydrogenase Hyf participate in acid tolerance by medium deacidification and facilitate urease activity, urinary stone formation and mouse colonization. A typical characteristic of *P. mirabilis* UTIs is the development of urinary stones. *P. mirabilis* would rapidly invade bladder epithelium cells and start to form extracellular clusters within the bladder lumen adjacent to the urothelium^38,39^. The concentrated urease activity within extracellular clusters would cause mineral deposition that serves as an early step in urinary stone formation^38^. Urease decomposes urea to produce ammonia, damaging renal epithelial cells^40^ and facilitating the formation of infectious urinary stones, carbonate apatite and struvite^38^. *P. mirabilis* extracellular clusters draw a massive neutrophil response and provide the environment to form biofilms^39^. The formation of extracellular clusters is critical to prevent clearance of bacteria by neutrophils and would influence the severity of infection^38,39^. In addition, medium deacidification has been shown to be involved in acid resistance of *Salmonella enterica* serovar Typhimurium^41^. We found FhlA and HyfG not only participated in medium deacidification and acid resistance (Figs. 1b, 3) but also affected urease activity and urinary stone formation in *P. mirabilis* (Fig. 4b). We identified that urease activity was pH-dependent, increasing in the pH range from 5 to 9 (Fig. 4a) and was dependent on FhlA and Hyf in the pH range from 5 to 8 (Fig. 4b). We showed that FhlA and Hyf-mediated deacidification or urine alkalization assisted in urease activity, stone formation and mouse colonization (Figs. 3a, 4b,c, 5), suggesting that FhlA and Hyf play a role in *P. mirabilis* virulence factor expression and virulence. In this regard, the data of transposon insertion-site sequencing (Tn-Seq) by Armbruster et al*.* have showed *P. mirabilis hyfB*, *hyfC*, *hyfD*, *hyfE*, *hyfF*, *hyfG, fdhF,* and *fhlA* are all likely to contribute to colonization in vivo^21^.

The published *Proteus* genomes including *P. mirabilis* HI4320, *P. mirabilis* BB2000, *P. hauseri*, *P. penneri* and *P. vulgaris* contain only a hydrogenase 4-like operon but not the hydrogenase 3-like operon. It is likely that hydrogenase 4-like operon is the functional counterpart of hydrogenase 3-like operon for encoding the protein complex joining with FDH-H to form FHL in *Proteus species.* The facts that PCR produced a product of 4228 bp but not the predicted 524 bp using *P. mirabilis* N2 genomic DNA as the template with primers annealing to the conserved region of *hycD* and *hycE* (data not shown) and *P. mirabilis hyfG* mutant displayed reduced survival against acid exposure (Fig. 1b) as the *hyc* mutant^18^ reinforce the notion. Hyf belongs to group 4a hydrogenase and is highly conserved in gammaproteobacteria^13^. The key role of Hyf is to convert proton to hydrogen, namely deacidification^11,13^. The structure of Hyf is similar to respiratory complex I, indicating Hyf is probably involved in the formation of proton gradient and energy production^13,19^. We found *hyfG* and *fhlA* are required for the full extent of acid tolerance in *P. mirabilis* (Fig. 1b). The dye DiOC_2_ commonly used to detect membrane proton gradient does not work in *P. mirabilis* N2 as is the case for *P. mirabilis* HI4320^33^. Instead, we determined other PMF-related phenotypes, motility and drug susceptibility, to know whether Hyf is involved in conservation of the proton gradient and PMF. No difference of swarming motility, swimming and drug susceptibility between wild-type strain and *hyfG* or *fhlA* mutant may indicate that the formation of PMF by FhlA-Hyf is negligible or compensable in these conditions. Although proton consumption should be the main reason for *fhlA* and *hyf*-involved acid tolerance. Possibly, it cannot be ruled out that the extent of PMF provided by FhlA-regulated Hyf may contribute to acid resistance of *P. mirabilis*. Recent studies showed that FHL-2 (FDH+Hyf) might couple with ATPase to generate PMF^42,43^ and formate affects ATPase activity and changes the number of thiol groups^43^. Hyf activity is ATP-dependent, while the formate can increase the F_0_F_1_-ATPase activity^42^. It is worth noting that the effect of formate on ATPase activity disappeared in *hyf* mutant or under the respiratory condition^44^. In addition, the formate dehydrogenase-H activity was dependent on Hyf and F_0_F_1_-ATPase^16^. These studies indicate FHL-2 may contribute to regulation of formate-associated ATPase under fermentative condition. In this regard, our finding that *P. mirabilis* Hyf did not play an important role in generating PMF under aerobic condition may attribute to coupling of ATPase with FHL-2 only under fermentative or formate-rich conditions. Thus, it could not be ruled out that Hyf is involved in PMF formation under oxygen-limited condition during infection and contribute to virulence.

Unlike *fhlA* and *hyfG* mutants, the urease activity of *rpoN* mutant still significantly decreased compared to the wild-type strain at pH 9 (Fig. 4b). Apparently, RpoN would have other regulatory pathways to affect urease activity at pH 9. In *P. mirabilis*, urease subunits and accessory proteins encoded by *ureDABCEFG* operon has been shown to be regulated positively by UreR and negatively by H-NS in the transcription level^45^. Previous study showed that RpoN and its cognate enhancer binding protein NtrC regulated expression of urease gene operon indirectly through control *nac*, encoding a transcriptional factor Nac which could activate urease gene operon in *K. pneumonia*^46^. We will investigate if other enhancer binding proteins such as NtrC, QseF and PspF are involved in the pathways of RpoN-regulated urease expression. In addition, we will examine whether UreR and H-NS are subject to the control of RpoN.

We found urinary stone formation ability of *rpoN* mutant was not impaired while *fhlA* mutant (lacking the enhancer binding protein, FhlA) was. In this regard, it was established that amino acids have effects on stone formation. For example, serine and asparagine could serve as the catalysts of this process^47^. Addition of asparagine did increase stone formation of wild-type *P. mirabilis* (data not shown). In addition, expression of asparagine synthetase gene (*asnA*) was downregulated by RpoN in wild-type *P. mirabilis* (data not shown). It is likely that loss of *rpoN* increases the asparagine level, compensating for the effect of *rpoN* loss.

This study showed *P. mirabilis fdhF* and *hyf* but not *fhlA* were induced significantly by formate and anaerobiosis respectively (Fig. 2e–g). Previous reports showed formate and anaerobiosis could trigger transcription of FhlA-regulated *hypABCDE*-*fhlA* operon in *E. coli*^10,26^. Two reasons could account for the discrepancy of *fhlA* expression in response to formate. First, In *E. coli*, *hypABCDE*-*fhlA* constitute an operon subject to the control of FhlA, namely self-regulation of FhlA^10^. This is not the case for *P. mirabilis*, whose *fhlA* promoter is not dependent on RpoN and FhlA (Fig. 2a). Second, formate serves as a ligand to activate FhlA, thus leading to transcription of FhlA-dependent genes including *hypABCDE*-*fhlA* operon in *E. coli*^48^ but not *fhlA* in *P. mirabilis*. As for anaerobic induction of *fhlA*, there are an FNR binding site in the promoter region and an OxyS-interacting site in the 5′UTR of *fhlA* in *E. coli*^26,49^. FNR is a global regulatory protein that regulates gene expression in response to oxygen deprivation in *E. coli* and it was shown that FNR acts as an anaerobic activator of *hypABCDE*-*fhlA* operon expression to control the expression of the hydrogenase maturation genes (Hyp)^26^ and FhlA. OxyS, a small regulatory RNA (sRNA) which is induced in response to the oxygen and oxidative stress in *E. coli*, inhibits *fhlA* translation by pairing with a short sequence overlapping the Shine-Dalgarno sequence, thereby blocking ribosome binding and translation^49^. Neither corresponding OxyS was found after searching for *P. mirabilis* sRNAs in the Bacterial Small Regulatory RNA Database (BSRD, https://kwanlab.bio.cuhk.edu.hk/BSRD/), nor FNR binding site was present in the *P. mirabilis fhlA* promoter region analyzed by RSAT. Therefore, unlike *E. coli fhlA* which is regulated by OxyS sRNA and transcription factor FNR sensing oxygen availability, the *P. mirabilis* promoter of *fhlA* was unresponsive to anaerobiosis (Fig. 2e).

This study implies that the proton-consuming acid resistance mechanism such as glutamate- and arginine-dependent acid resistance^50^ may also contribute to urease activity in the presence of glutamate or arginine. Our preliminary data showed urease activity of wild-type *P. mirabilis* was induced by arginine and glutamate. What is the biological significance of the proton-consuming acid resistance of FHL? The different metabolites or signals would trigger different acid resistance system. Under stationary phase or the presence of extracellular glutamate, glutamate-dependent acid resistance would be important to bacterial acid response^51^. Likewise, arginine-dependent acid resistance is triggered by extracellular arginine^50^. However, FHL is the amino acid-independent acid resistance induced by formate or anaerobiosis^50^. In this study, we found that *fdhF*, *hyfG* and *fhlA* of FHL genes of wild-type *P. mirabilis* were expressed when cultured in the aerobic condition (Fig. 2b–d) and the expression of *fdhF* and *hyf* could also be induced by formate or anaerobiosis (Fig. 2f,g).

**Figure 7:**
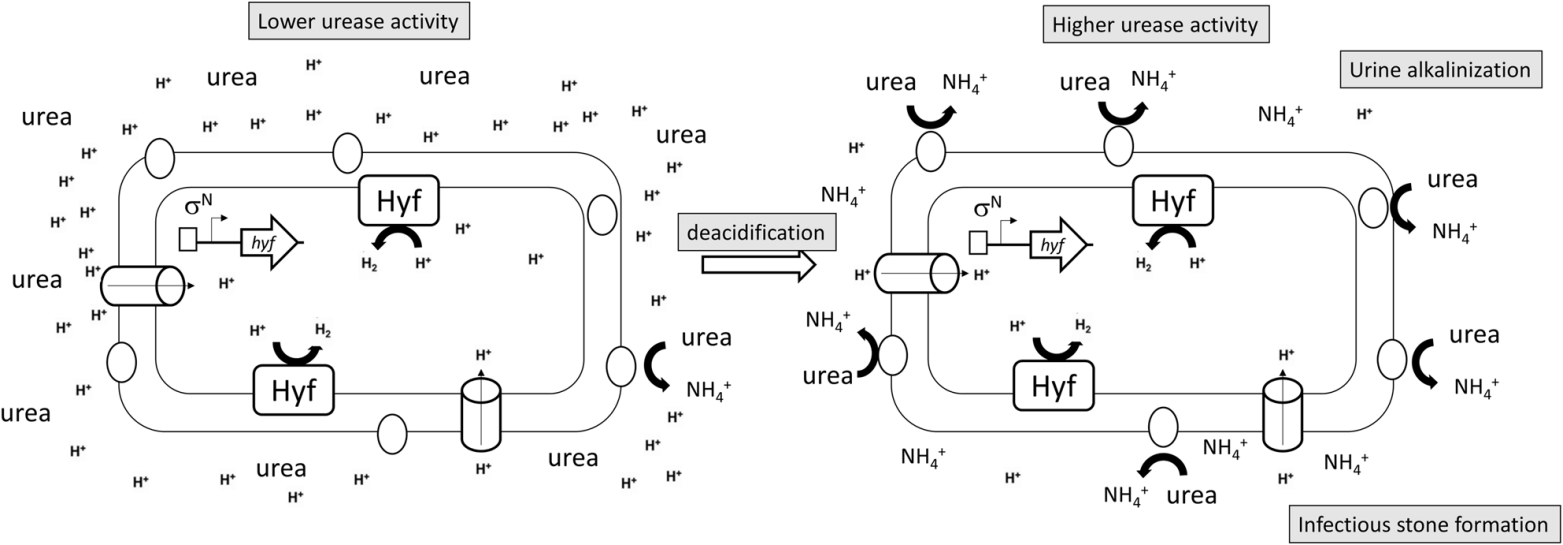
Putative FhlA-dependent Hyf expression accelerates deacidification to facilitate urease activity and urinary stone formation during the early stage of urinary tract infection. *hyf* operon expression requires RpoN (σ^N^) and the FhlA activator. (Left) When bacteria enter the urinary tract of about pH 5.0, the urease activity is limited. Deacidification by FhlA-dependent Hyf increases urease activity to rise the urine pH value. (Right) Higher urine pH leads to higher urease activity further alkalizing urine to facilitate infectious urinary stone formation. Rectangle, FhlA; oval, urease; cylinder, proton channel; a right-angled arrow, RpoN-dependent transcription start.

In this study, we showed FhlA-dependent *hyf* expression accelerates deacidification to facilitate urease activity and infectious urinary stone formation of *P. mirabilis* (Fig. 7) and subsequently mouse colonization. This is the first report investigating how *P. mirabilis* FHL-associated genes are involved in virulence and linking the ability of acid resistance to urease activity of *P. mirabilis*. The formate-induced FHL pathway provides a perspective for development of new approaches to counteract *P. mirabilis* UTIs. The prohibition against food producing formate could be a preventive measure to combat *P. mirabilis* UTIs. For example, uptake of aspartame increases urine formate level^52^, which could trigger FHL system to facilitate urease activity and subsequently urinary stone formation and in vivo colonization. In addition, our findings support the notion that manipulation of the urine pHn (the pH above which calcium and magnesium phosphates come out of solution in urine) could form the basis of a strategy to prevent catheter encrustation in those with urinary tract colonization by urease-positive bacteria^53^.

## Materials and methods

### Bacterial strains, plasmids, primers, reagents and growth conditions

The bacterial strains, plasmids and primers used in this study are listed in Tables 1 and 4. All chemicals were obtained from the Sigma-Aldrich unless otherwise indicated. Bacteria were routinely cultured in Luria–Bertani (LB) broth at 37 ℃ with shaking at 200–250 rpm. The LSW^−^ agar ^54^ was used to prevent the phenotypic expression of swarming motility for selecting mutant clones and determining CFU. Ampicillin (100 μg/ml), chloramphenicol (40 μg/ml), kanamycin (100 μg/ml), tetracycline (20 μg/ml), streptomycin (100 μg/ml) was added to the medium as needed.

**Table 4:** Strains and plasmids.

Strain or plasmid	Genotype or relevant phenotype	Source or reference
***P. mirabilis***		
wt	*P. mirabilis* N2; wild-type strain; Tc^r^	Clinical isolate
*rpoN*	wt derivative; *rpoN*-knockout mutant; Km^r^	This study
*fhlA*	wt derivative; *fhlA*-knockout mutant; Km^r^	This study
*hyfG*	wt derivative; *hyfG*-knockout mutant; Km^r^	This study
*fhlA*c	*fhlA* mutant containing pGEM-T Easy-*fhlA*; Amp^r^ Km^r^	This study
*hyfG*c	*hyfG* mutant containing pGEM-T Easy-*hyfG*; Amp^r^ Km^r^	This study
*fhlA*cSD	*fhlA* mutant containing pGEM-T Easy-*fhlA*(F291I, T292S); Amp^r^ Km^r^	This study
**Plasmids**		
pGEM-T Easy	TA cloning vector; Amp^r^	Promega
pGEM-T Easy-*rpoN*	pGEM-T Easy containing intact *rpoN* sequence including its RBS; Amp^r^	This study
pGEM-T Easy-*fhlA*	pGEM-T Easy containing intact *fhlA* sequence including its RBS; Amp^r^	This study
pGEM-T Easy-*hyfG*	pGEM-T Easy containing intact *hyfG* sequence including its RBS; Amp^r^	This study
pGEM-T Easy-*fhlA*(F291I,T292S)	pGEM-T Easy containing an altered FhlA with the conserved RpoN-interacting motif GAFTGA changing into GAISGA; Amp^r^	This study
pACYC184-*fhlA-xylE*	*fhlA* reporter plasmid, pACYC184 containing intact *fhlA* promoter sequence before *xylE*; Cm^r^	This study
pACYC184-*fdhF-xylE*	*fdhF* reporter plasmid, pACYC184 containing intact *fdhF* promoter sequence before *xylE*; Cm^r^	This study
pACYC184-*hyf-xylE*	*hyf* reporter plasmid, pACYC184 containing intact *hyf* operon promoter sequence before *xylE*; Cm^r^	This study

Synthetic urine was prepared according to a recipe previously described^27,28^ containing the following components (g/l): CaCl_2_·2H_2_O, 0.651; MgCl_2_·6H_2_O, 0.651; NaCl, 4.6; Na_2_SO_4_, 2.3; sodium citrate, 0.65; sodium oxalate, 0.02; KH_2_PO_4_, 2.8; KCl, 1.6; NH_4_Cl, 1.0; urea, 25.0; creatine, 1.1 and tryptic soy broth, 10.0. pH was adjusted to 5.8 and urine was sterilized by passing through a 0.2 μm pore-size filter.

The anaerobiosis was established and maintained in the anaerobic chamber (Whitley A35 anaerobic workstation, 5% H_2_–5% CO_2_–90% N_2_).

### Acid resistance assay

Acid resistance assay was performed as described by Wu et al.^55^ with some modifications. Overnight bacterial cultures were diluted 500-fold with fresh LB broth in centrifuge tubes and incubated at 37 °C with shaking at 220 rpm for 4 h. Cells were incubated in LB medium at pH 3.0 (± 0.1) for 2 h. Acid-treated cells and the untreated control (cells before the acid treatment) then were washed with phosphate-buffered saline (PBS), serially diluted in PBS and plated on LSW^−^ agar plates to determine the CFU. Acid survival rate (expressed as percent) was calculated with the following formula: 100 × (CFU after acid treatment/CFU before acid treatment).

### Swarming assay

The swarming assay was performed on LB agar (tryptone, 10 g/l; yeast extract, 5 g/l; NaCl, 0.5 g/l; agar, 1.5%, w/v) plates as described previously^30^. The swarming migration distance was monitored by following swarm fronts of the bacterial cells and recording progress every hour.

### Swimming assay

The swimming assay was performed on LB agar (agar, 0.3%, w/v) plates as described previously^30^. The swimming migration distance was recorded at 18 h after inoculation.

### MIC assay

MICs of antibiotics for wild-type and mutant strains were determined by the broth microdilution method according to the guidelines proposed by the Clinical and Laboratory Standards Institute.

### Growth curve analysis

Bacteria were grown overnight at 37 ℃, the cultures were diluted to an initial optical density at 600 nm (OD_600_) of 0.01 in LB with or without 7.5 μM CCCP and then the OD_600_ values were measured at 1-h intervals up to 8 h with a spectrophotometer.

### Urease activity (phenol-hypochlorite) assay

Phenol-hypochlorite assay is based on the detection of ammonia released during urea hydrolysis. Ammonia can react with phenol-hypochlorite at high pH to form blue indophenol^56^. Bacteria grown to log phase (5 h after subculture) were adjusted to 2 × 10^9^ CFU using LB and incubated statically in LB containing urea at 0.5 M for 2 h at 37 ℃. Supernatants were collected (70 μl per well of 96-well microplates) for urease activity measurement after centrifugation at 12,000 rpm for 5 min. Urease activity was determined by measuring ammonia production after 2-h incubation in LB containing urea at 0.5 M. Briefly, 70 μl of phenol reagent (2%(w/v) phenol in 75% ethanol) and 70 μl of alkali reagent (0.28 M sodium hydroxide and 100 mM sodium hypochlorite) were added to each well. After one hour, the absorbance at 625 nm was measured using a microplate reader (SpectraMax M2, USA).

### Urinary stone formation assay

Urinary stone formation assay was performed as described previously^27,28^ with some modifications. Wild-type and mutant strains were grown overnight in LB broth and diluted 100-fold in the same medium. After incubation for 5 h, bacteria were adjusted to 2 × 10^8^ CFU/ml with synthetic urine and incubated for another 1 h in the static condition at 37 ℃. The optical density at 630 nm of well-suspended suspensions was measured as the intensity of stone formation.

### UTI mouse model

The mouse model of UTIs was used as described previously^57^. Six- to eight-week old female ICR mice were injected transurethrally with overnight cultures of bacteria at a dose of 1.5 × 10^7^ CFU per mouse. On day 3 after injection, mice were sacrificed and bladder and kidney samples were collected to determine the viable bacterial count. All animal experiments were performed in strict accordance to the recommendations in the Guide for the Care and Use of Laboratory Animals of the National Laboratory Animal Center (Taiwan), and the protocol was approved by the Institutional Animal Care and Use Committee of National Taiwan University College of Medicine.

### Reporter assay

The reporter plasmid-transformed wild-type and mutant strains were grown overnight in LB broth with chloramphenicol (40 μg/ml) and diluted (100-fold) into 10 ml LB broth. Promoter activity was measured as described previously^58^. For monitoring expression profiles, the XylE activity was monitored at 3, 5, 7 and 24 h after incubation. For signal induction, overnight bacterial cultures were regrown for 5 h and 30-min induction was performed in different conditions before the XylE activity was measured.

### Deacidification assay

Overnight cultures were diluted 100-fold in fresh LB medium and incubated at 37 °C with shaking at 220 rpm for 5 h. Bacterial pellet was resuspended in synthetic urine (pH 4.8 ± 0.2). The pH value was monitored during 30–90 min at 10-min intervals by a pH meter (Jencomodel 6173, USA).

### Statistical analysis

Statistical analyses were performed using GraphPad Prism software, version 6.01 or Microsoft Excel 2016. The specific statistical tests used are described in the figure legends.
